# Inflammatory Arthritis and Bone Metabolism Regulated by Type 2 Innate and Adaptive Immunity

**DOI:** 10.3390/ijms23031104

**Published:** 2022-01-20

**Authors:** Yasunori Omata, Michael Frech, Taku Saito, Georg Schett, Mario M. Zaiss, Sakae Tanaka

**Affiliations:** 1 Department of Orthopaedic Surgery, Faculty of Medicine, The University of Tokyo, 7-3-1 Hongo, Bunkyo-ku, Tokyo 113-0033, Japan; yasunoriomata1024@g.ecc.u-tokyo.ac.jp (Y.O.); tasaitoutky@g.ecc.u-tokyo.ac.jp (T.S.); 2 Bone and Cartilage Regenerative Medicine, Graduate School of Medicine, The University of Tokyo, 7-3-1 Hongo, Bunkyo-ku, Tokyo 113-0033, Japan; 3 Department of Internal Medicine 3, Rheumatology and Immunology, Friedrich-Alexander-University Erlangen-Nürnberg (FAU) and Universitätsklinikum Erlangen, Ulmenweg 18, 91054 Erlangen, Germany; michael.frech@fau.de (M.F.); georg.schett@uk-erlangen.de (G.S.); mario.zaiss@uk-erlangen.de (M.M.Z.); 4 Deutsches Zentrum Immuntherapie, Friedrich-Alexander-University Erlangen-Nürnberg (FAU) and Universitätsklinikum Erlangen, Ulmenweg 18, 91054 Erlangen, Germany

**Keywords:** type 2 immunity, rheumatoid arthritis, osteoclast, type 2 innate lymphoid cells

## Abstract

While type 2 immunity has traditionally been associated with the control of parasitic infections and allergic reactions, increasing evidence suggests that type 2 immunity exerts regulatory functions on inflammatory diseases such as arthritis, and also on bone homeostasis. This review summarizes the current evidence of the regulatory role of type 2 immunity in arthritis and bone. Key type 2 cytokines, like interleukin (IL)-4 and IL-13, but also others such as IL-5, IL-9, IL-25, and IL-33, exert regulatory properties on arthritis, dampening inflammation and inducing resolution of joint swelling. Furthermore, these cytokines share anti-osteoclastogenic properties and thereby reduce bone resorption and protect bone. Cellular effectors of this action are both T cells (i.e., Th2 and Th9 cells), but also non-T cells, like type 2 innate lymphoid cells (ILC2). Key regulatory actions mediated by type 2 cytokines and immune cells on both inflammation as well as bone homeostasis are discussed.

## 1. Introduction

Host immune responses are exerted by innate and adaptive immune cells. Both innate immune responses and antigen-specific adaptive responses are essential in defending against pathogens and controlling infections, and also contribute to chronic inflammation and bone loss. While traditionally, the role of type 1 immune responses has been investigated in terms of their role in inflammation and bone, recent studies revealed that type 2 immunity has regulatory properties on a disease like arthritis, and also affects bone metabolism.

**Figure 1:**
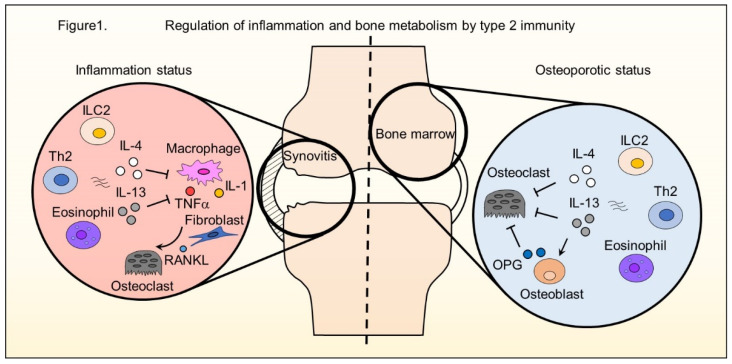
Regulation of inflammation and bone metabolism by type 2 immunity. In an inflammatory status of RA, ILC2s, Th2 cells, and eosinophils exert anti-inflammatory properties in the inflamed joint by producing type 2 cytokines such as IL-4 and IL-13. Type 2 cytokines suppress the production of inflammatory cytokines by macrophages. Activated macrophages secrete proinflammatory cytokines IL-1 and TNFα, which stimulate synovial fibroblasts to express RANKL. Type 2 immunity can modulate bone metabolism by regulating the secretion of inflammatory cytokines. In the bone marrow environment, osteoclasts and osteoblasts regulate bone metabolism. IL-4 and IL-13, which are secreted by type 2 immune cells, control osteoclast differentiation directly and indirectly.

Classically, type 1 immunity and type 2 immunity are differentiated. Type 1 immunity comprises T helper 1 (Th1) cells, type 1 innate lymphoid cells (ILC1s), and natural killer (NK) cells, which induce cytotoxicity and phagocytosis by secreting interferon gamma (IFNγ). Type 2 immunity is exerted by T helper 2 (Th2) cells, eosinophils, mast cells, basophils, Th9 cells, and the recently discovered type 2 innate lymphoid cells (ILC2s) (Figure 1). A third type of immunity is characterized by Th17 cells and type 3 innate lymphoid cells (ILC3s). These immune cells communicate with each other and regulate inflammation to adjust and balance immune responses [1]. In inflammatory diseases such as rheumatoid arthritis (RA) demonstrating proliferative synovitis and bone destruction, innate and adaptive immunity are involved in the pathology [2]. Activated macrophages secrete interleukin (IL)-1, IL-6, and tumor necrosis factor (TNF)α, which stimulate synovial fibroblasts to express receptor activator of NF-κB ligand (RANKL) in RA [3]. Th17 cells secrete IL-17 to stimulate macrophages and synovial fibroblasts [2]. As RANKL is a key molecule for osteoclast differentiation, bone metabolism shifts toward bone destruction in the inflammatory status. Recent studies uncovered the regulation of arthritis and bone metabolism by Type 2 immune cells (Figure 1).

**Figure 2:**
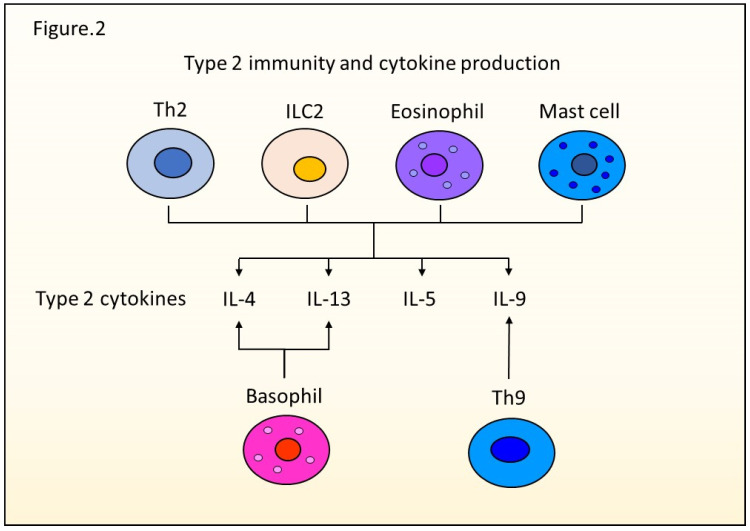
Type 2 immunity and cytokine production. Th2 cells, ILC2s, eosinophils, mast cells, basophils, and Th9 cells are type 2 immune cells producing IL-4, IL-5, IL-9, and IL-13.

Dendritic cells (DCs) initially prime and stimulate naïve T cells, and CD4+ T cells differentiate into Th2 cells expressing the GATA-binding protein 3 gene (GATA-3) [4,5]. Th2 cells can produce type 2 cytokines such as IL-4, IL-5, IL-9, and IL-13. Several immune cells such as Th2 cells, Th9 cells, eosinophils, mast cells, and basophils are known to exert type 2 immunity by producing those cytokines (Figure 2). Type 2 immunity has two major functions: defending against helminth infection by enhancing barrier function and protecting against autoimmune diseases [6,7,8]. For instance, IL-4 and IL-13, key mediators of type 2 immunity, are essential to neutralize worms, such as the gastrointestinal parasite, *Nippostrongylus brasiliensis*. Although this worm was expelled in IL-4 knockout mice, IL-4 receptor chain-deficient mice and Stat6-deficient mice failed to expel it. IL-13, rather than IL-4, is essential to expel the worm via Stat6 signaling [6].

Besides infection control, type 2 immunity controls mucus production by Goblet cells in the intestine, thereby contributing to protective barriers. A decrease in IL-4 production in the intestinal lamina propria is observed in inflammatory bowel disease, reducing barrier function and promoting inflammation [7]. Type 2 immunity also plays roles in asthma, atopic dermatitis, and other allergic reactions [9]. Asthma and other allergic reactions promote the production of mucus.

With regard to adaptive immunity, IL-4-producing Th2 cells migrate into lymphoid tissues and differentiate into follicular helper T (TFH) cells to support immunoglobulin synthesis and isotype switching [10]. IL-4 promotes the differentiation of Th2 cells and induces B cells to produce immunoglobulin E (IgE). IL-5 and IL-9 recruit eosinophils and evoke eosinophilic inflammation [11]. Type 1 immunity produces interferon-gamma (IFNγ), IL-12, and IL-18, which act to suppress type 2 immunity.

Although T cells are the major source of those type 2 cytokines, it was found that Rag2 knockout mice can also produce these cytokines [12]. As Rag2 knockout mice lack T cells and B cells, this suggested the existence of other Th2 cytokine sources. Progress in type 2 immunology arose from the discovery of innate lymphoid cells (ILCs) [13,14,15,16]. ILCs do not have antigen receptors. Several groups of ILCs were discovered, and they were differentiated and characterized by transcriptional profiling and functions such as cytokine production. For instance, IL-4-, IL-5-, and IL-13- producing lineage (−) c-Kit (+) Sca-1 (+) lymphocytes reside in gut fat tissue [13,14,15,16]. Neil et al. found nuocytes producing type 2 cytokines in mesenteric lymph nodes by analyzing IL-13-reporter mice [14]. Aside from infection control and barrier function, the cells and cytokines associated with type 2 immunity also exert homeostatic functions, and ILCs exert immune homeostatic effects [17]. This review summarizes the homeostatic effects of type 2 immunity in arthritis and bone metabolism, highlighting the role of type 2 cytokines and ILC2s.

## 2. Type 2 Immune Responses in Arthritis

**Figure 3:**
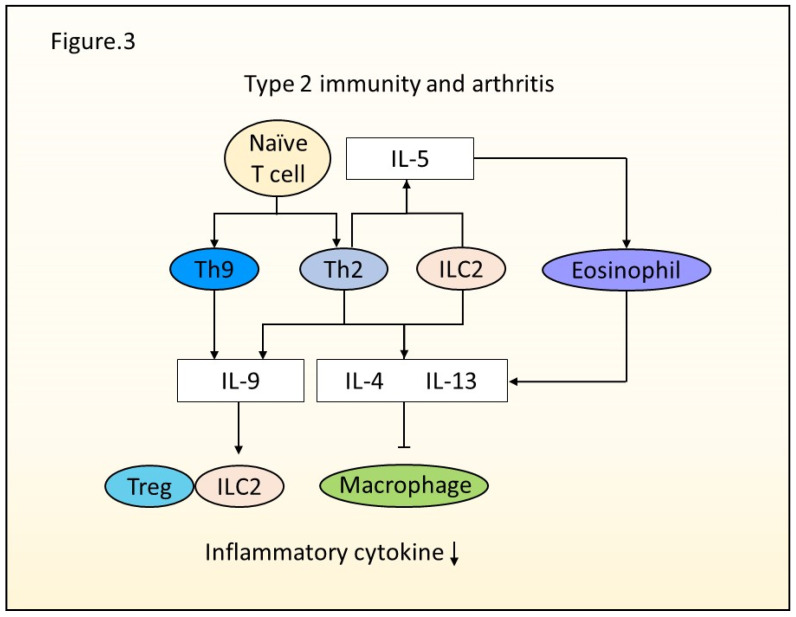
Type 2 immunity and arthritis. Naive T cells differentiate into Th2 cells by the activation of GATA3 genes. Th2 cells and ILC2s produce IL-4 and IL-13, which suppress macrophage activation in the inflammatory status. Th2 cells and ILC2s also secrete IL-5, activating eosinophils to generate IL-4 and IL-13. Naive T cell-derived Th2 and Th9 can produce IL-9. IL-9 enhances the interaction of ILC2s and Tregs to alleviate inflammation. ILC2s regulate both the initiation and resolution of arthritis.

RA is a chronic autoimmune disease, with joint inflammation inducing destruction of joint at the bare area. Many factors, such as genetic and environmental factors, are involved in the initiation and the pathology of RA. Genetic studies revealed the involvement of genetic factors in RA and a high rate of coincidence in monozygotic twins [18]. Environmental factors like smoking, preexisting pulmonary diseases, and microbial dysbiosis are associated with RA development [19]. RA is characterized by joint inflammation involving many immune cells, such as fibroblasts, macrophages, T cells, and B cells. Type 1 immunity protects from pathogens and develops arthritis by the pro-inflammatory effect of IL-1β and IFNγ. IL-18 and IL-15, which are dominant in the synovial fluid of RA patients, can induce Th1 cells, ILC1s, and NK cells [20,21]. IL-1β stimulates synoviocytes and chondrocytes to produce extracellular matrix degradative enzymes such as matrix metalloproteinases (MMPs), and it also regulates the formation of osteoclasts by stimulating osteoblasts to produce RANKL [22]. The efficacy of a recombinant human IL-1Ra, Anakinra, has been established in the treatment of active RA patients [23]. Although type 1 immunity modulates arthritis, the existence of another T cell subset was suggested in the 2000s. Microbial stimulation induced IL-17 in both mouse and human T cells [24]. Indeed, a murine study using genetic deletions of IL-12 and IFNγ has still shown the development of autoimmune responses [25]. IL-17 producing T helper cells (Th17) were found as a discrete T cell subset exerting a pathogenic role in arthritis [26]. While type 2 immunity has long been neglected in arthritis, there is increasing evidence for the role of type 2 cytokines and cells in arthritis (Figure 3).

Interleukin-4. IL-4 has anti-inflammatory effects on human monocytes and macrophages and suppresses the production of TNFα and IL-1β [27,28]. The study of polymorphism in 108 early RA patients revealed the polymorphic IL-4 gene sequence located at 5q31-31 [29]. Furthermore, IL-4-590 promoter polymorphism was observed and was suggested as a genetic risk factor for the severity of RA [30,31,32,33]. IL-4R single-nucleotide polymorphism (SNP) is associated with radiographic progression in RA patients treated with methotrexate (MTX) monotherapy [34]. In experimental arthritis animal models, IL-4 treatment reduced the progression of arthritis, including collagen-induced arthritis (CIA), proteoglycan-induced arthritis (PIA), and methylated BSA/IL-1-induced arthritis [35,36,37]. In the CIA model, continuous administration of IL-4 delayed the onset of arthritis with less severe joint damage and inflammation [35]. In the PIA model, IL-4 knockout mice exhibited an increased frequency of arthritis [36]. This exacerbation of arthritis was suggested to involve STAT6 signaling. Methylated BSA/IL-1-induced arthritic mice treated with neutralizing IL-4 antibody showed a 30% reduction in arthritis, suggesting IL-4 regulates CD4 T cell-dependent arthritis [37]. The severity of arthritis was exacerbated in IL-4Rα knockout mice and LysM^cre^IL-4Ra^flox/−^ mice [38]. IL-4 has an anti-angiogenic property that suppresses vascular endothelial growth factor (VEGF) expression in synovial fibroblasts [39]. Helminth infection by *Nippostrongylus brasiliensis,* which robustly induced Th2 cells and accumulated eosinophils, reduced a TNFtg arthritis mice model by IL-4/IL-13-induced STAT6 signaling [40]. On the other hand, Th1 immunity exerts inflammation, and the differentiation of Th1 is mediated by IL-12 via STAT4 expression [41].

Interleukin-25. Type 2 immunity is triggered by IL-25, IL-33, and thymic stromal lymphopoietin (TSLP). IL-25 induces type 2 cytokines such as IL-4, IL-5, and IL-13 with blood eosinophilia [12]. IL-25 belongs to the IL-17 family, and the signal transduces through a heterodimeric receptor of IL-17 receptor A and B. IL-25 and IL-17A share the same receptor chain for the downstream signaling; it was shown that IL-25 exerts its anti-inflammatory property by acting as an antagonist competing with IL-17A [42]. The level of IL-25 was increased in the plasma of RA patients, and the expression of IL-25 in synovium was induced belatedly after the stimulation of TNFα and IL-17A. In the mice arthritis model, type 2 collagen-induced arthritis, the level of IL-25 was elevated in the late stage of arthritis, while IL-17 was increased in the early stage [43]. IL-25 suppressed the expression level of IL-17 and induced IL-4. IL-25 is exerted from activated eosinophils and basophils and enhances the function of the Th2 cell-producing cytokines IL-4, IL-5, and IL-13 [44]. Intriguingly, IL-25 treatment in Rag knockout mice led to the production of IL-5 and IL-13 [12], suggesting the existence of non-T cells that respond to IL-25. This evidence led to the discovery of ILCs.

Interleukin-33. IL-33 is an IL-1 family cytokine and, as with IL-25, is a main producer of type 2 cytokines. IL-33 is produced by epithelial cells, endothelial cells, DCs, macrophages, and fibroblasts. IL-33 drives and activates type 2 immunity, involving cells including Th2 cells, ILC2s, and mast cells. IL-33 is expressed in RA patients’ synovial fibroblasts [45]. The serum IL-33 level in RA patients is higher during high disease activity than low and moderate disease activity [46]. While mice deficient in the IL-33 Receptor, ST2, exhibited attenuation of arthritis [45,47], the severity of arthritis in the IL-33 knockout mice was similar to wild-type control mice. However, an in vitro assay showed that bone resorption activity was higher in the IL-33 knockout mice compared to wild-type mice in a collagen-induced arthritis model. The administration of IL-33 to TNF transgenic mice led to these mice reducing cartilage destruction and bone loss [48].

Interleukin-13. IL-13 is a ubiquitous cytokine with diverse functions in allergy and inflammation [49,50]. IL-13 transduces the signal by binding to IL-4Rα and IL-13Rα2. As IL-4 also uses IL-4Rα for signal transduction through STAT6, similarities exist between the functions of IL-4 and IL-13 [51]. Phosphorylated STAT6 translocates into the nucleus and binds to the promoter regions of responsive genes. IL-13 is a dominant and pathogenic property in inflammation in eosinophilic disorders such as asthma, atopic dermatitis, and eosinophilic esophagitis. On the other hand, IL-13 has an anti-inflammatory effect on macrophages by inhibiting inflammatory cytokines such as TNFα and IL-1β [52]. In CIA mice, the severity and the incidence of arthritis were suppressed by the treatment of IL-13 [53], and IL-13 exhibited anti-inflammatory properties on arthritis. The study of RA polymorphism in IL-13, including IL-4 and IL-4Rα, did not show genetic association [54], while in another study, IL-13 polymorphism was observed in the RA disease progressive group [55].

Interleukin-5. IL-5 and its receptor induce the migration of eosinophils. Lung eosinophilia induces severe asthma by promoting IL-5 signaling. Recent clinical progress has been made by the discovery of biological agents targeting IL-5/IL-5R, such as Mepolizumab, Benralizumab, and Reslizumab [56]. Randomized control studies (RCTs) revealed the efficacy of these agents against severe eosinophilic asthma [57,58,59]. Levels of IL-5 and eotaxin in the serum of RA patients were higher than in healthy control subjects [60,61]. IL-5 secreted from Th2 cells enhances IL-13 production by eosinophils and promotes macrophage polarization from the M1 to M2 phenotype [62]. In arthritis models, IL-5 is expressed in the arthritic joint synovium [8], and eosinophilic infiltration at the local joint modulated the resolution of arthritis by IL-5 [39]. Overexpression of IL-5 and transfer of IL-5 in K/BxN arthritic mice reduced the inflammation of joints. IL-5 is a potent anti-inflammatory mediator through its modulation of eosinophils.

Interleukin-9. A pleiotropic function of IL-9 regulates various immune cells by binding to a receptor consisting of IL-9 receptor α- chain and γc-chain. IL-9 has dual roles in the pathogenesis of immune diseases [63]. IL-9 is produced by ILC2 and Th9 cells, which are differentiated by IL-4, transforming growth factor (TGF)-β, and TSLP [64,65,66]. It induces Th2 cell-mediated responses with mucus production in the epithelial cells. IL-9 is up-regulated before the clinical onset of RA and is overexpressed in the synovial tissues [67]. IL-9 is also increased in the lungs of patients with asthma, where it promotes allergic inflammation [68]. A recent study discovered that ILC2s are one of the major sources of IL-9 production in arthritis, and that they regulate the resolution of arthritis [69]. The level of IL-9 and the frequency of IL-9-producing CD3^+^ T cells in RA patients’ synovial fluid is higher than in the synovial fluid of OA patients [70], while IL-9 serum levels have not been found as a reliable marker for disease activity in RA [71].

## 3. Type 2 Innate Lymphoid Cells in Arthritis

ILCs are composed of five subsets, ILC1-3, NK cells, and lymphoid tissue inducer (LTi) cells. They are differentiated from common lymphoid progenitors (CLPs), and share their functions with T cells, as they produce similar cytokines. ILCs have tissue-specific functions regulating biological homeostasis and diverse diseases [16]. ILC1s share their cytokines with Th1 cells, producing IL-12, IL-15, and IL-18. ILC1s also share the same transcriptional factor, T-bet. ILC2s produce type 2 cytokines IL-4, IL-5, and IL-13, with the transcriptional expression of GATA3 and RORα. ILC3s are differentiated by the expression of RORγτ, and produce IL-17, IL-22, and GM-CSF. ILCs exhibit their plasticity and show crosstalk with T cells [72]. Of the ILCs, ILC2s generate a type 2 property by producing type 2 cytokines. While they initiate allergic inflammation, such as lung asthma [73], recent studies have revealed that ILC2s effectively attenuate arthritis [69]. The induction of IL-4/IL-5/IL-13(+) ILC2s mitigated arthritis, suggesting the inflammation is regulated by ILC2s. In vitro assays showed that ILC2s affected LPS-induced macrophages, and that IL-4(+)/IL-13(+) ILC2s suppressed the production of IL-1β and TNFα [6]. ILC2s differentiate from CLPs by stimulating IL-25 and IL-33 and producing Th2 cytokines such as IL-4, IL-5, IL-9, and IL-13. In arthritic conditions, numbers of ILC2s were increased according to the progression of arthritis. Rauber et al. investigated the role of IL-9(+) ILC2s in regulating arthritis at the resolution phase. IL-9(+) ILC2s promote the resolution of arthritis by interfering with regulatory T cells (Tregs) [69]. Regarding the effect of ILC2s on the initiation phase of arthritis, IL-4(+)/IL-5(+)/IL-13(+) ILC2 numbers are increased from the beginning of arthritis in mice. In RA patients, the disease activity score was inversely correlated with the frequency of ILC2s. Stier et al. [74] reported that injection of IL-33 or the administration of pulmonary fungal allergen mobilized ILC2 progenitors to exit the bone marrow. Consistent with this result, the production of bone marrow ILC2s preceded their increase in synovial tissues in arthritis [75]. RORγ and Gata3 are essential transcription factors in the production of ILC2. RORα^cre^/Gata3^fl/fl^ mice that exhibited a significantly reduced frequency of ILC2 showed an increased number of osteoclasts in bones. In contrast, overexpression models of ILC2, using IL-25/IL-33 mini-circle vectors, IL-2/IL-2 antibody complex, and adoptive transfer of ILC2, exhibited an increase in bone volume with a decreased number of osteoclasts. The technology of hepatocyte-specific mini-circle vectors with tail hydrodynamic injection enables systemic overexpression of encoding proteins [76]. Adoptive transfer of adequate ILC2s to arthritis models, in which the isolated cells were expanded in vitro through stimulation using cytokines such as IL-2, IL-7, IL-25, IL-33, and TSLP [77], mitigated experimental arthritis. These studies indicate that ILC2 could regulate arthritis by modulating type 2 immunity with IL-4 and IL-13 at the initial phase and with IL-9 at the resolution phase of the condition. The different properties of ILC2s between the conditions of allergy and arthritis could be explained by the differing effects type 2 cytokines exhibit in respective pathological conditions.

Type 2 immunity may also be involved in colitis and other forms of arthritis, such as spondyloarthritis (SpA). IL-33 activated ILC2 during *Clostridium difficile* infection, inhibiting colitis [78]. ILC2s and Tregs also mitigated dextran sulfate salt (DSS)-induced colitis in mice by recovering the epithelial barrier dysfunction and shifting macrophage polarization toward the M2 phase [79,80]. In psoriatic arthritis (PsA) patients, circulating ILC2s were decreased [81], while IL-17/IL-22-expressing ILC3s were increased. The ILC2/ILC3 ratio correlated with the activity and the remission of the disease. With respect to SpA, it has been shown that IL-4/IL-13 receptors are enriched at the peri-entheseal bone site, and the signaling axis regulates entheseal IL-23 production [82]. The evidence suggests a, most likely regulatory, interaction between type 2 immune cells and IL-17 producing cells.

## 4. Type 2 Immunity and Bone Metabolism

**Figure 4:**
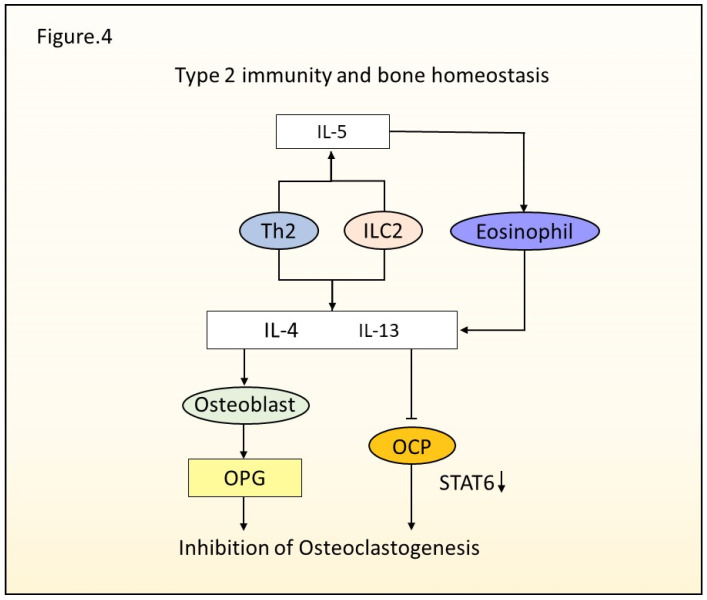
Type 2 immunity and bone homeostasis. Th2 cells and ILC2s generate IL-4, IL-5, and IL-13. IL-5 activates eosinophil, and it secretes IL-4 and IL-13. Type 2 cytokines suppress the differentiation of osteoclasts by regulating the expression of STAT6. Type 2 cytokines stimulate osteoblasts to generate OPG, which inhibits osteoclastogenesis.

Bone consists of bone-resorbing osteoclasts, bone-forming osteoblasts, and structural osteocytes. Osteoblasts are mononucleated cells derived from mesenchymal stem cells, while osteoclasts are multinucleated cells originating from hematopoietic progenitor cells. Bone remodeling, in which the matrix produced by osteoblasts repair resorbed bone surfaces is essential for maintaining bone homeostasis [83]. Osteoporosis is caused by the age-related imbalance of hormones, inflammation, or tumorigenesis. Bone turnover is determined by the balance of osteoclasts and osteoblasts, with a shift toward bone resorption occurring in osteoporosis. Accelerated bone resorption leads to fragility and insufficiency fractures. The balance between osteoclasts and osteoblasts is adjusted by the RANKL/RANK/OPG system. Osteoprotegerin (OPG) binds to RANKL and blocks the binding of RANKL to RANK, which leads to the inhibition of osteoclast differentiation. Single nucleotide polymorphism in *TNFRSF11B*, which encodes OPG, is associated with postmenopausal osteoporosis [84,85,86]. The involvement of immune cells in bone biology has been investigated. Human and murine Treg cells inhibit osteoclast differentiation [87,88]. It was suggested that the suppression of osteoclasts was mediated by direct cell–cell interactions or in an indirect cytokine-dependent manner. It has been uncovered that various immune cells, including T cells, B cells, and innate immune cells, regulate bone metabolism and homeostasis. This section provides an overview of the crosstalk that occurs between bone metabolism and type 2 immunity, with a focus on the type 2 effector cytokines (Figure 4).

Interleukin-4. IL-4 has an inhibitory effect on RANKL- and TNFα-induced osteoclast formation in vitro in a dose-dependent manner [89,90,91,92,93,94,95]. IL-4 targets early osteoclast precursors and suppresses NFκB activation and mitogen-activated protein kinase (MAPK) signaling in a STAT6-dependent manner [93,96]. IL-4 also inhibits resorption pit formation in cultures of bone marrow macrophages with RANKL or TNFα. IL-4 loses its ability to inhibit osteoclastogenesis in the cells of IL-4R knockout mice [95]. As the coculture of IL-4R knockout spleen cells with wild-type T cells inhibited osteoclast formation, the inhibition of osteoclastogenesis by IL-4 depends on T-cell activation. On the other hand, lymphocyte-specific IL-4 transgenic mice (*lck*-IL-4 transgenic mice) exhibited osteoporosis by decreasing osteoblast-induced bone formation [97]. In histological sections, the *lck*-IL-4 transgenic mice showed a significant decrease in osteoclast-associated TRAP activity.

Watanabe et al. demonstrated that IL-4 reduces bone resorption in an in vitro assay using a coculture system [89]. IL-4 exerts the inhibitory effect on osteoclasts by several cascades. IL-4 modulates NFκB, JNK, p38, ERK, and TNFα signaling, and it inhibits NFκB and MAPK activation via STAT6 signaling [93,94]. A study of DCs showed IL-4 to be more effective than IL-13 for the differentiation and function of DCs [98]. In osteoclastogenesis, IL-4 potently inhibits osteoclast differentiation compared to IL-13 [99]. IL-4 induces more rapid phosphorylation of STAT6 than IL-13, while the induction of the production of OPG, which inhibits osteoclast differentiation, was equivalent between IL-4 and IL-13.

Interleukin-25. In vitro analysis by Min et al. has shown pretreatment of IL-25 suppressed RANKL-induced human osteoclast differentiation in a dose-dependent manner and decreased the expression level of the osteoclast markers NFATc1 and Cathepsin K [100]. They also identified the effect of IL-25 in an arthritic condition and showed that IL-25 suppressed IL-22-induced osteoclast formation. As osteoclast precursors express IL-17 receptors in murine monocytes [101], IL-25 may antagonize osteoclast differentiation.

Interleukin-33. IL-33 increases osteoblast differentiation [102]. IL-33 inhibits RANKL-induced osteoclast differentiation [48], and mice lacking the IL-33 receptor ST2 (*Il1rI1*-deficient mice) exhibit normal bone formation but enhanced bone resorption [103]. IL-33 induced some pro-apoptotic molecules in osteoclasts, such as BAX (Bcl2-associated X protein), FAS, and FASL (FAS ligand). Osteoclast activity is higher in IL-33 knockout mice than in wild-type control mice [47]. On the other hand, it has been reported that IL-33 promotes osteoclastic bone resorption via TRAF6 in human CD4+ monocytes. As IL-33 and IL-25 induced ILC2 [8], it was suggested that IL-33 regulated bone homeostasis indirectly and directly and, therefore, is involved in in vivo regulation [75].

Interleukin-13. IL-13 shares similarities with IL-4 [49,104]. In osteoblastogenesis, IL-13 has an inhibitory effect on osteoblasts through suppression of ALP activity. IL-13 inhibits bone resorption by suppressing prostaglandin synthesis in osteoblasts. In human osteoblast culture, IL-13 inhibits the proliferation and production of IL-6 [105,106]. IL-13 and IL-4 share receptor components and exert similar effects in human osteoblastogenesis. IL-13 induces OPG, a soluble decoy receptor that inhibits RANKL and osteoclastic resorption [107].

Interleukin-5. IL-5 is involved in the development of eosinophils in the bone marrow and regulates the late differentiation of eosinophils [108,109]. Eosinophils produce the type 2 cytokine IL-4, which regulates bone metabolism [110]. A transgenic mouse line overexpressing IL-5 demonstrated an increase in eosinophils and progenitor cells [111], and induced marrow-derived osteoprogenitor cells and IL-5-mediated suppression of the recruitment of osteoclasts in the mediation of bone formation [112]. IL-5 regulates bone metabolism indirectly via eosinophils or directly by modulating osteogenic progenitors and inhibiting osteoclast recruitment.

## 5. ILC2 and Bone Homeostasis

Type 2 cytokines not only exhibit anti-inflammatory properties in arthritis but also regulate osteoclast differentiation. IL-4- and IL-13-producing ILC2s have the potential to modulate bone homeostasis by regulating osteoclast differentiation [75]. Adoptive transfer of ILC2s decreases osteoclast numbers, and ILC2s migrate into bones. A histometric bone analysis revealed that the transferred ILC2s could recover the decreased bone volume by ovariectomy (OVX) in mice. Interestingly, the adoptive transfer of IL-4(−)/IL-13(−) ILC2s fails to rescue the reduced bone volume induced by OVX. Furthermore, ILC2s reside in the proximity of osteoclasts, suggesting an interaction with ILC2s. ILC2s also suppress RANKL-induced osteoclastogenesis in vitro. The adoptive transfer of IL-4/IL-13-competent ILC2s induces an increase in Tregs, suggesting an indirect effect on bone homeostasis via Tregs. A direct association between Tregs and ILC2s has been demonstrated [113]. T cell-producing IL-2 drives ILC2 cytokine production, including IL-4 and IL-5. Importantly, Tregs protect from arthritic joint destruction and systemic bone loss. Serum levels of bone resorption markers, such as TRAP-5b and CTX-1, inversely correlate with the frequency of circulating Tregs in healthy controls and RA patients [114].

Tie2^cre^RORγ^fl/fl^ mice, which are the model of endothelial-specific RORγ deletion and exhibit a significantly reduced frequency of ILC2s, showed decreased bone volume with increased osteoclast number. The addition of ILC2s reduced osteoclast differentiation and resorption pit formation. Furthermore, IL-4(−)/IL-13(−) ILC2s did not show the suppressive effect observed in the condition with IL-4/IL-13 competent ILC2s. A supernatant coculture media of ILC2s and bone marrow-derived macrophages (BMMs) showed a reduction in osteoclast formation. Interestingly, this suppression was rescued by adding IL-4(−)/IL-13(−) ILC2s, indicating the effect of cytokines secreted by ILC2s. Furthermore, the addition of IL-4/IL-13-competent ILC2s to STAT6-deficient BMMs failed to suppress osteoclast differentiation. Thus, ILC2s regulate bone homeostasis via cytokine secretion and cell interaction with T cells, thereby controlling osteoclast differentiation.

## 6. Conclusions

In summary, type 2 immunity regulates arthritis and bone homeostasis via Th2 cells, ILC2s, and other type 2 response-related immune cells. Type 2 cytokines released from the adaptive and innate immune cells have synergistic properties both in vivo and in vitro in arthritis and in bone homeostasis in both steady-state and pathological conditions. Type 2 immune cells have pleiotropic effects on immunity in local tissues, including bone marrow and joints. However, the effects of the modulation of these immune cells on bone and joint metabolism are still only partly understood. Further investigation of the homeostasis regulated by type 2 immunity will provide new insights and unveil the intricate mechanisms of pathological conditions such as osteoporosis and arthritis.

## Data Availability

Not applicable.
